# The serine protease homolog *spheroide* is involved in sensing of pathogenic Gram-positive bacteria

**DOI:** 10.1371/journal.pone.0188339

**Published:** 2017-12-06

**Authors:** Jelena Patrnogic, Vincent Leclerc

**Affiliations:** UPR9022 du CNRS, Institut de Biologie Moleculaire et Cellulaire, Universite de Strasbourg, Strasbourg, France; Biomedical Sciences Research Center Alexander Fleming, GREECE

## Abstract

In *Drosophila*, recognition of pathogens such as Gram-positive bacteria and fungi triggers the activation of proteolytic cascades and the subsequent activation of the Toll pathway. This response can be achieved by either detection of pathogen associated molecular patterns or by sensing microbial proteolytic activities (“danger signals”). Previous data suggested that certain serine protease homologs (serine protease folds that lack an active catalytic triad) could be involved in the pathway. We generated a null mutant of the serine protease homolog *spheroide (sphe)*. These mutant flies are susceptible to *Enterococcus faecalis* infection and unable to fully activate the Toll pathway. Sphe is required to activate the Toll pathway after challenge with pathogenic Gram-Positive bacteria. Sphe functions in the danger signal pathway, downstream or at the level of Persephone.

## Introduction

The fruit fly, *Drosophila melanogaster*, spends its life among decaying matter and rotten fruit, where it coexists with different microorganisms. One of the main characteristics of the immune response of *Drosophila melanogaster* is the challenge-induced synthesis and secretion of antimicrobial peptides (AMPs). This response involves the activation of two signal transduction cascades–the Toll and IMD pathways [1]. Gram-positive bacteria and fungi activate the Toll pathway, whereas Gram-negative bacteria and Gram-positive bacteria of the genus *Bacillus* activate the IMD pathway [2]. In both cases, signaling leads to the activation of NF-*κ*B transcription factors and expression of target genes including AMPs.

In the late 1980’s, Charles Janeway proposed that the innate immune mechanisms are essential for the early detection and defense against infection. These mechanisms discriminate between self and microbial non-self. Janeway proposed the existence of germ-line encoded pathogen recognition receptors (PRRs) that recognize conserved signature molecules expressed by pathogens, referred to as Pathogen Associated Molecular Patterns (PAMPs) [3]. A few years later, Polly Matzinger proposed the danger signal hypothesis. This hypothesis proposed that the activation of immune mechanisms is not due to discrimination between self and non-self, but rather to sensing of danger signals: either recognition of pathogens, or alarm signals produced by microbial activities or by the host’s own damaged cells or tissues [4].

The Toll pathway can be activated in two ways: recognition of PAMPs by circulating Pathogen Recognition Receptors (PRRs) in the hemolymph or by virulence factors, mostly proteases, secreted by the pathogens. This activation triggers proteolytic cascades in the hemolymph. The terminal protease in the cascade cleaves Spaetzle to its activated ligand form, which is able to bind the Toll receptor and activate the intracellular pathway. Depending on the triggering signal, two proteolytic cascades can be distinguished. First, the recognition cascade activated by PAMPs which includes 3 serine proteases, ModSP [5], Grass [6, 7] and SPE [8]. Second, the danger signal cascade can be activated by pathogen-encoded, secreted proteases. Such abnormal protease activity indicates that potentially dangerous changes are happening. Danger signaling involves the serine protease Persephone (Psh) [6].

There are over two hundred genes coding for serine proteases (SPs) and serine protease homologs (SPHs) in the *Drosophila* genome [9]. SPHs maintain the serine protease fold but lack amidase activity since at least one of the catalytic triad residues is missing [9]. The physiological functions of SPHs are poorly understood, although they have been implicated in different arthropod immune responses, in the horseshoe crab [10], *Manduca sexta* [11] and *Anopheles gambiae* [12, 13].

We identified the protease Grass as being required for Toll pathway activation downstream of PRRs [6]. Grass was initially identified during an RNAi based screen of serine proteases and serine protease homologs [7], but its function was incorrectly assigned, probably due to the incomplete knockdown of the gene mediated by RNAi. We decided to verify the function of the other candidates identified in this work and focused on the serine protease homolog Spheroide (Sphe). It has been reported that Sphe is involved in the activation of Toll pathway. The knockdown of *sphe* by RNAi induced the same phenotype upon immune challenge as that of SPE, implying that Sphe might function as an adaptor or regulator of SPE [7].

Here, we use a null mutant of *sphe* to demonstrate that Sphe is involved in the activation of Toll pathway. By using protease-deficient bacteria we conclude that Sphe is sensing the virulence factors (proteases) produced by pathogenic Gram-positive bacteria. Furthermore, using flies that are double mutants for both *sphe* and *grass*, we show that Sphe is involved in the danger signal cascade.

## Results

### Sphe is required in the activation of the immune response after a challenge with *Enterococcus faecalis*

As previously shown for *Grass*, RNAi mediated knock-down could potentially give different results compared to null mutants [6]. The reasons for this are not always clear and could be attributed to incomplete knock-down and OFF-target effects that are not detected by bioinformatics tools. To circumvent limitations of RNAi-mediated knockdown, we looked for another way of inactivating *sphe*. In the fly line *Mi{ET1}spheroide*^*MB11555*^ a Minos transposon element is inserted 412 bp downstream of the start codon, in an intronic sequence. This insertion reduced the expression of the *sphe* transcript compared to wild type (S1A Fig). Flies in which *sphe* expression was reduced were more susceptible than wild type flies to infection with the Gram-positive bacterium *Enterococcus faecalis* (S1C Fig). *Sphe* mutants showed reduced activation of the Toll pathway after immune challenge compared to wild type flies as measured by the levels (30%) of *drosomycin (drs)* antimicrobial peptide gene expression (S1B and S1D Fig).

We excised the Minos insertion element to confirm that the susceptibility phenotype was due to its insertion in *sphe*. We obtained a line with precise excision of the element (*sphe*^*Δ11*^) that expresses wild type *sphe* mRNA levels. When *sphe*^***Δ****11*^ flies were challenged with *Enterococcus faecalis* they showed normal expression of *drs* (S1A and S1B Fig). This excision line is used as a wild type control in subsequent experiments (*ctrl*) unless otherwise stated. This demonstrates that the *Minos* insertion was indeed responsible for the susceptibility phenotype. We also obtained two imprecise excisions, *sphe*^***Δ****49*^ and *sphe*^***Δ****104*^, in which no *sphe* expression was detected. The *sphe*^***Δ****49*^ deletion includes the entire transcript as well as 974 bp upstream that include 46 bp of the 3’UTR of *CG9673*, and 307 bp downstream that include the 5’UTR of *CG9676* (S1I Fig). The *sphe*^***Δ****104*^ deletion starts at the *Minos* insertion site and includes 835 bp of upstream sequence. At the protein level, the first 74 amino acids residues are missing, which include the signal peptide and 50 amino acids residues of the catalytic domain, including the His residue from the catalytic triad. Both deletions are therefore null alleles of *sphe*.

**Fig 1:**
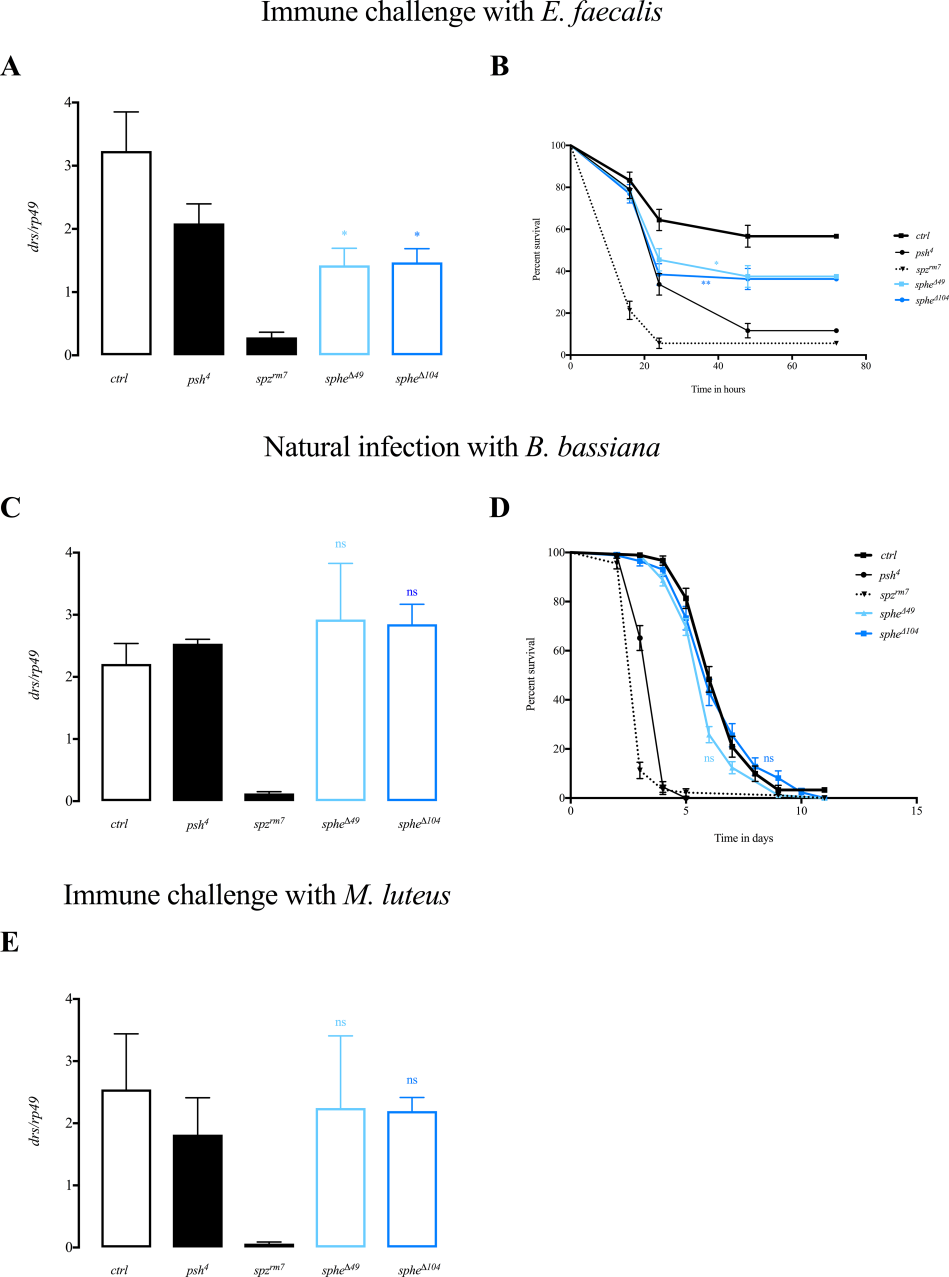
Sphe is involved in activation of immune response after a challenge with *E*. *faecalis*. *drosomycin* expression 24 hours PI, normalized to *rp49* after immune challenge with *E*. *faecalis* (*ctrl* (*sphe*^***Δ****11*^ wild type flies) vs. *sphe*^***Δ****49*^ p<0.0261, *ctrl* vs. *sphe*^***Δ****104*^ p<0.02) (A) or after natural infection with *B*. *bassiana* 48 hours PI (C) or immune challenge with *M*. *luteus* (E). Survival rate after immune challenge with *E*. *faecalis* (*ctrl* vs. *sphe*^***Δ****49*^ p<0.032, *ctrl* vs. *sphe*^***Δ****104*^ p<0.0065) (B) or natural infection with *B*. *bassiana* (D).

When *sphe* null mutant flies were challenged with pathogenic Gram-positive bacterium *Enterococcus faecalis* we observed a significant decrease of *drs* levels 24 hours after infection compared to that of wild type flies (*drs* reaches 45% of wild type level) (Fig 1A). Furthermore *sphe* flies are more susceptible to this immune challenge than wild type flies (Fig 1B). Since both null alleles *sphe*^***Δ****49*^ and *sphe*^***Δ****104*^ show the same phenotype, we will describe only the results obtained with *sphe*^***Δ****104*^.

When *sphe* null mutant flies, were challenged with the non-pathogenic Gram-positive bacterium, *Micrococcus luteus*, or by natural infection with the entomopathogenic fungus *Beauveria bassiana*, *drs* expression was comparable to that in wild type flies (Fig 1C and 1E and S1E–S1G Fig). Accordingly, *sphe* null mutant flies showed the same susceptibility to *Beauveria bassiana* infection as wild type flies (Fig 1D).

**Fig 2:**
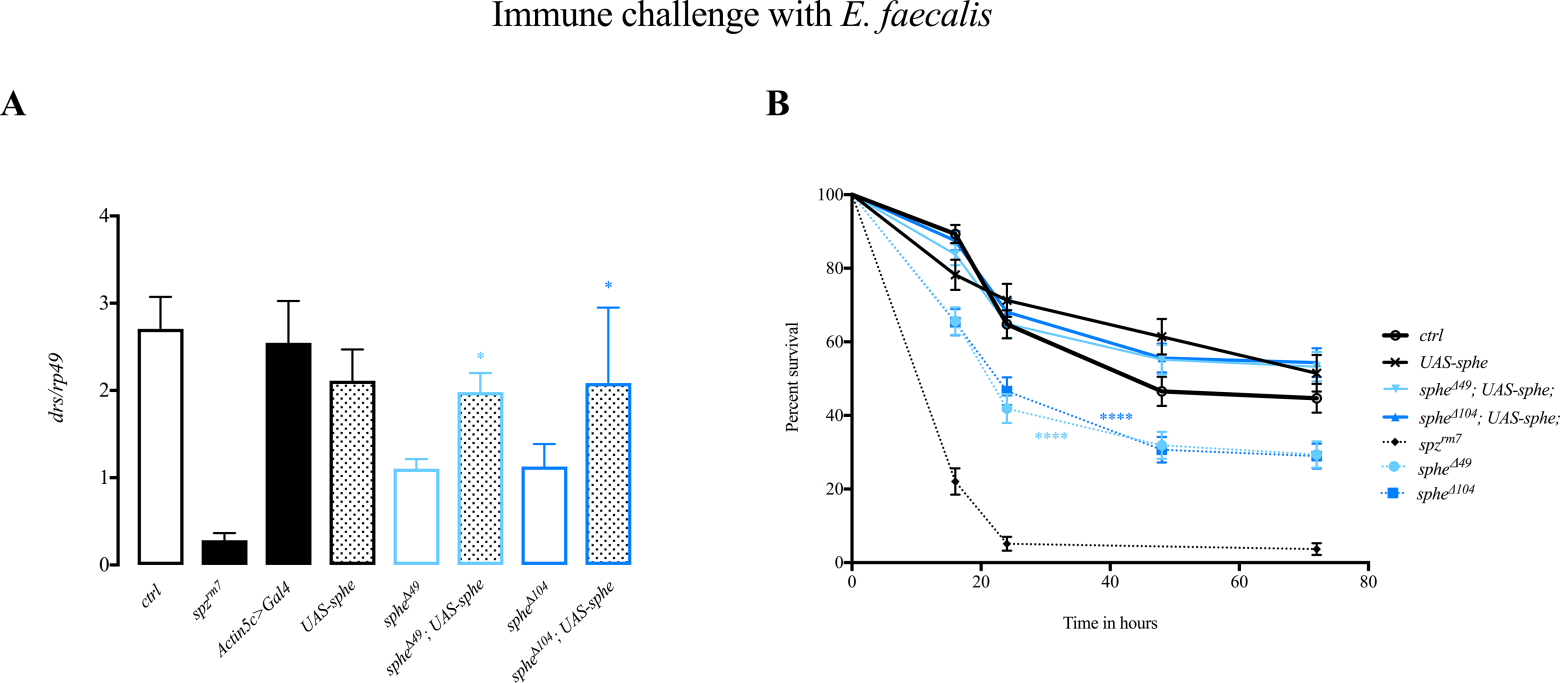
Rescue of *sphe* mutant phenotype using Gal4/UAS system. (A) *drosomycin* expression 24 hours PI, normalized to *rp49*, after immune challenge with *E*. *faecalis* (*sphe*^***Δ****49*^ vs. *sphe*^***Δ****49*^; *UAS-sphe* p<0.0103, *sphe*^***Δ****104*^ vs. *sphe*^***Δ****104*^;*UAS-sphe* p<0.0229). Controls (crtl) are *w*^*1118*^ wild type flies. (B) Survival rate after immune challenge with *E*. *faecalis* (*sphe*^***Δ****49*^ vs. *sphe*^***Δ****49*^; *UAS-sphe* p<0.0001, *sphe*^***Δ****104*^ vs. *sphe*^***Δ****104*^;*UAS-sphe* p<0.0001). For over expression of *sphe* using *UAS-sphe* lines we used ubiquitous *Act5C>Gal4*.

To confirm that the phenotype we observed is due to *sphe* inactivation, we overexpressed Sphe with the *UAS-Gal4* system using the ubiquitous *Actin5C>Gal4* driver. Sphe overexpressing flies are viable and show no obvious phenotype. Sphe overexpression does not induce the Toll pathway as measured by levels of *drs* mRNA. We therefore expressed Sphe in *sphe* mutant background, and observed the rescue of the phenotype as assayed by the induction of *drs* expression in response to *Enterococcus faecalis* infection, as well as an enhanced survival to the infection (Fig 2A and 2B).

Taken together, these data show that Sphe is involved in Toll pathway activation and is required to activate a full and efficient response to the pathogenic Gram-positive bacterium *Enterococcus faecalis*.

### Sphe is involved in the “danger” signal Toll activation cascade

**Fig 3:**
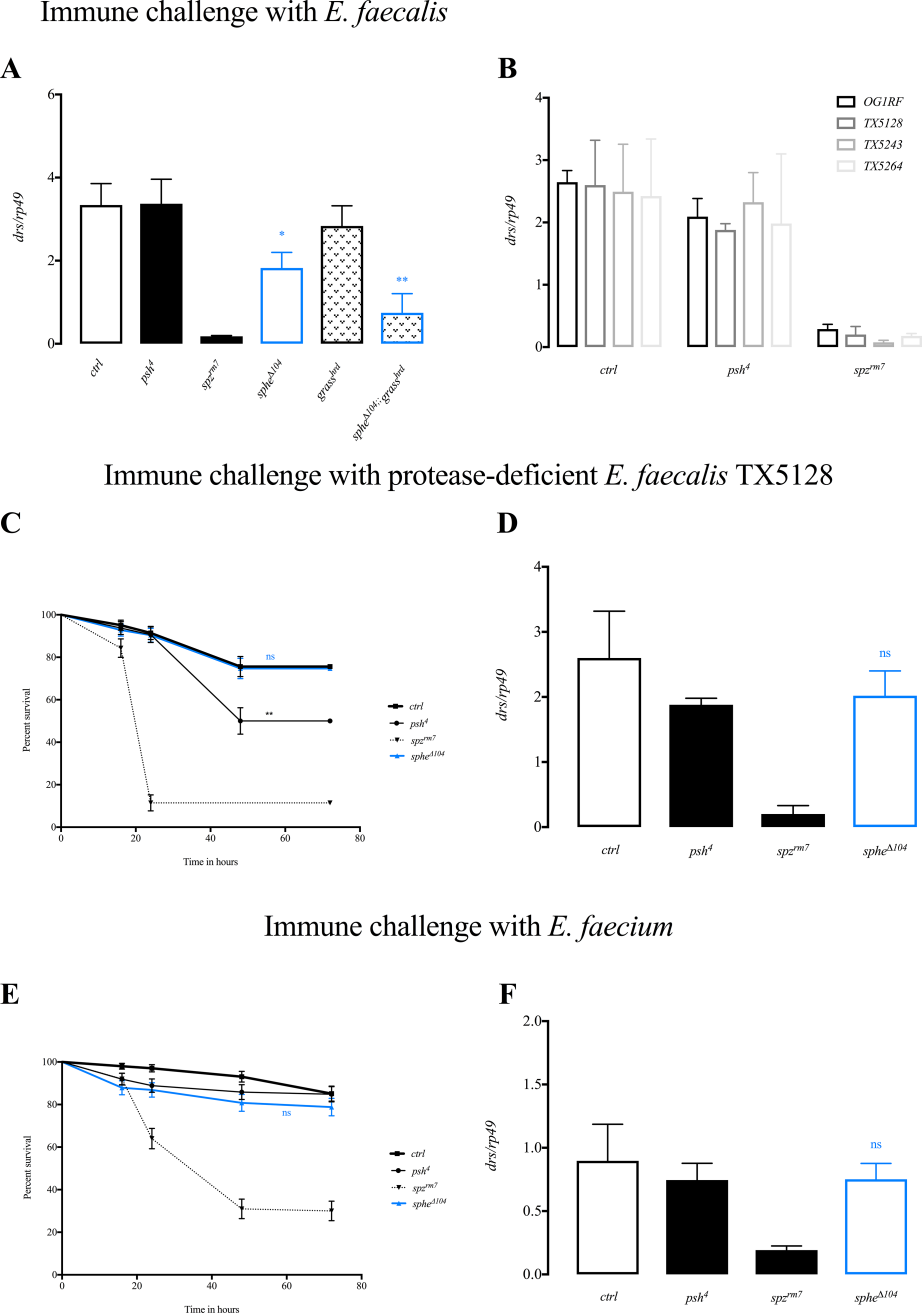
Sphe is involved in danger signal cascade. *drosomycin* expression 24 hours PI, normalized to *rp49* after immune challenge with *E*. *faecalis* (* *ctrl (sphe*^***Δ****11*^*)* vs. *sphe*^***Δ****104*^ p<0.0381, ** *sphe*^***Δ****104*^vs. *sphe*^***Δ****104*^*;;grass*^*hrd*^ p<0.0329) (A), after immune challenge with wild type and protease-deficient bacteria (comparison) (B) with protease-deficient strain of *E*. *faecalis* TX5128 (D) or with *E*. *faecium* (F); Survival rate after immune challenge with protease-deficient strain of *E*. *faecalis* TX5128 (**ctrl vs *psh4* p<0.0031. There are no explanations for this slight phenotype) (C), or with *E*. *faecium* (E).

*Enterococcus faecalis* is a pathogenic Gram-positive bacterium that activates the Toll pathway via both proteolytic branches, through recognition of Lys-type peptidoglycan [14], as well as through production of virulence factors that activate the “danger” signal cascade [6]. To assess in which of these branches Sphe is functioning, we generated double mutants *sphe*^***Δ****104*^*;grass*^*hrd*^ in which the recognition cascade is blocked and we challenged these flies with *Enterococcus faecalis*. The levels of *drs* expression 24 hours after immune challenge are significantly decreased (*drs* reaches 20% of wild type level) compared to both *sphe* and *grass*^*hrd*^ single mutants, to a level comparable to that of *spz* mutants flies (Fig 3A). This additive effect indicates that Sphe is acting in a parallel pathway to Grass, in the “danger” signal Toll activation cascade.

*Enterococcus faecalis* produces several virulence factors, including cytolysin, aggregation substance, the zinc metalloprotease gelatinase GelE, and the serine protease SprE [14, 15]. We focused on the secreted extracellular proteases GelE and SprE as potential virulence factors that might be sensed by the danger signal cascade. To confirm the involvement of *sphe* in this danger signal sensing, we used protease-deficient strains of *Enterococcus faecalis* that were mutant for either *gelE* (TX5264), *sprE* (TX5243), or both *gelE* and *sprE* (TX5128) [16, 17]. We observe slight, but reproducible, reductions in *drs* levels when wild type flies are challenged with protease-deficient bacteria compared to those challenged with wild type bacteria. The observed decrease in *drs* levels is similar to the one observed in *psh* mutant flies challenged with wild type bacteria suggesting that these proteases are required for activating the danger signal pathway. This is confirmed with the fact that there is no additive effect when *psh* mutant flies are challenged with protease-deficient bacteria compared to the same infection in wild type flies (Fig 3B).

After protease-deficient bacteria immune challenge, *sphe* mutants behave as wild type flies and show no susceptibility to the protease-deficient bacteria (Fig 3C) and the levels of *drs* 24 hours after infection are as in wild type controls indicating normal activation of Toll pathway (Fig 3D). The same result was found using either of the single mutants for *gelE* or *sprE* (S2A and S2B Fig) indicating that both of these virulence factors contribute to the activation of Toll pathway. We confirmed this observation by using the non-pathogenic bacterium *Enterococcus faecium* that is closely related to *E*. *faecalis* but lacks these virulence factors [18]. After immune challenge with *E*. *faecium*, *sphe* mutant flies show no susceptibility (Fig 3E) and *drs* levels 24 hours after infection are as in wild type controls indicating normal activation of Toll pathway (Fig 3F). Taken together these data demonstrate that Sphe is involved in sensing proteases produced by *Enterococcus faecalis* for Toll pathway activation.

### Sphe is involved in sensing Gram-positive pathogenic bacteria

**Fig 4:**
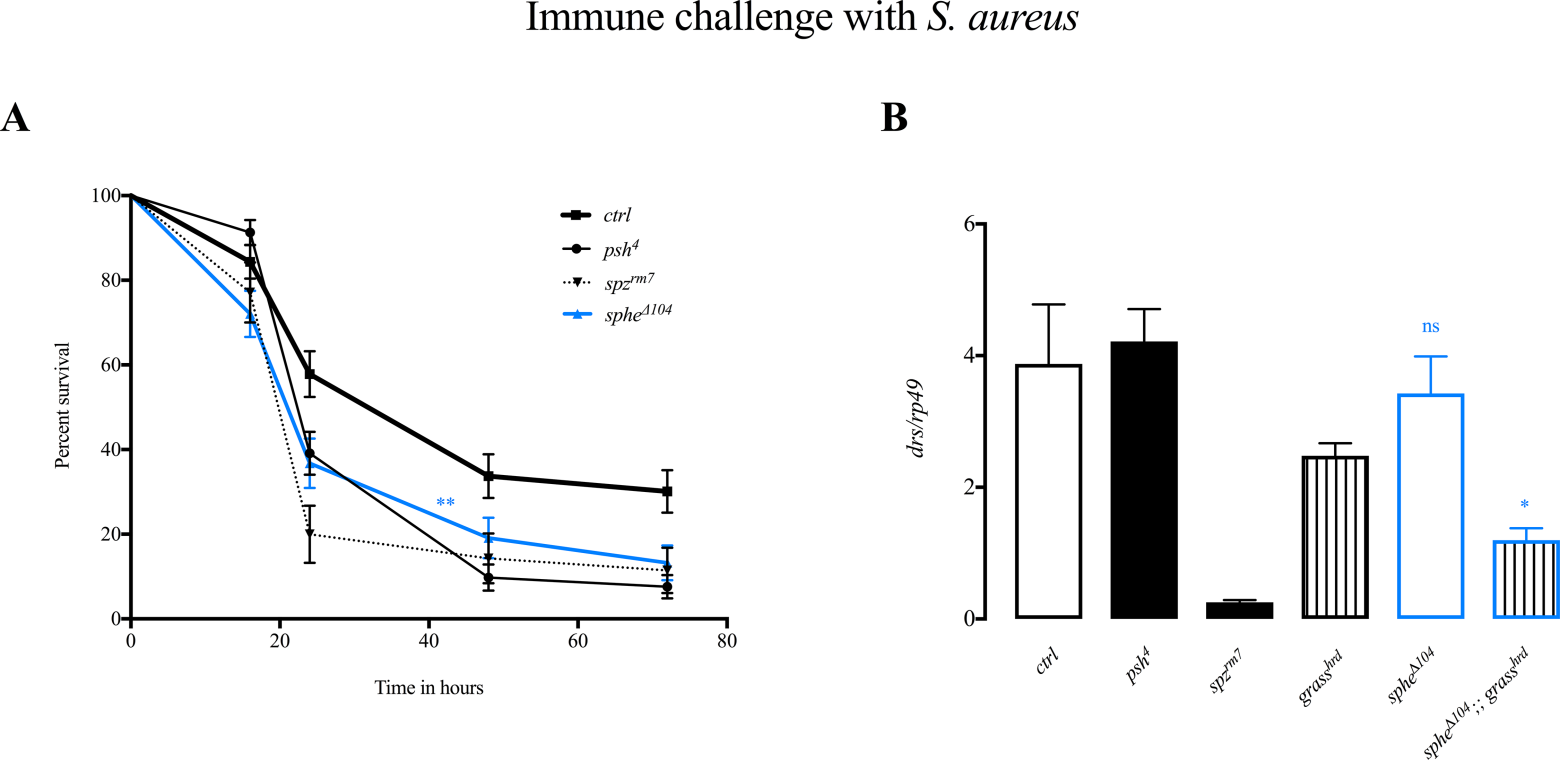
Sphe is involved in sensing *S*. *aureus*. Immune challenge with *S*. *aureus*: survival rate (*ctrl* vs. *sphe*^*Δ*104^ p<0.0038) (A); *drosomycin* expression 24 hours PI, normalized to *rp49* (*sphe*^*Δ*104^ vs. *sphe*^***Δ****104*^*;;grass*^*hrd*^ p<0.0144) (B). *sphe*^***Δ****11*^ wild type flies are used as control (ctrl).

We tested another pathogenic Gram-positive bacterium, *Staphylococcus aureus*. We observed that *sphe* null mutant flies showed the same susceptibility as *psh* mutant flies to infection compared to wild type flies (Fig 4A), but *drs* levels 24 hours upon immune challenge are as in wild type controls (Fig 4B). The wild type activation of Toll pathway could however be due to the PRR pathway. In order to confirm the involvement in the “danger” signal cascade, we used double mutant *sphe*^***Δ****104*^*;grass*^*hrd*^ flies in which the recognition cascade is also blocked. *drs* levels are significantly decreased in *sphe*^***Δ****104*^*;grass*^*hrd*^ double mutants compared to the levels in wild type flies and to both *sphe* or *grass*^*hrd*^ single mutant flies (it reaches 30% of wild type flies) (Fig 4B). These data demonstrate that Sphe and Grass act in parallel in the sensing of virulence factors produced by *Staphylococcus aureus*.

### Sphe is acting in the Persephone pathway

**Fig 5:**
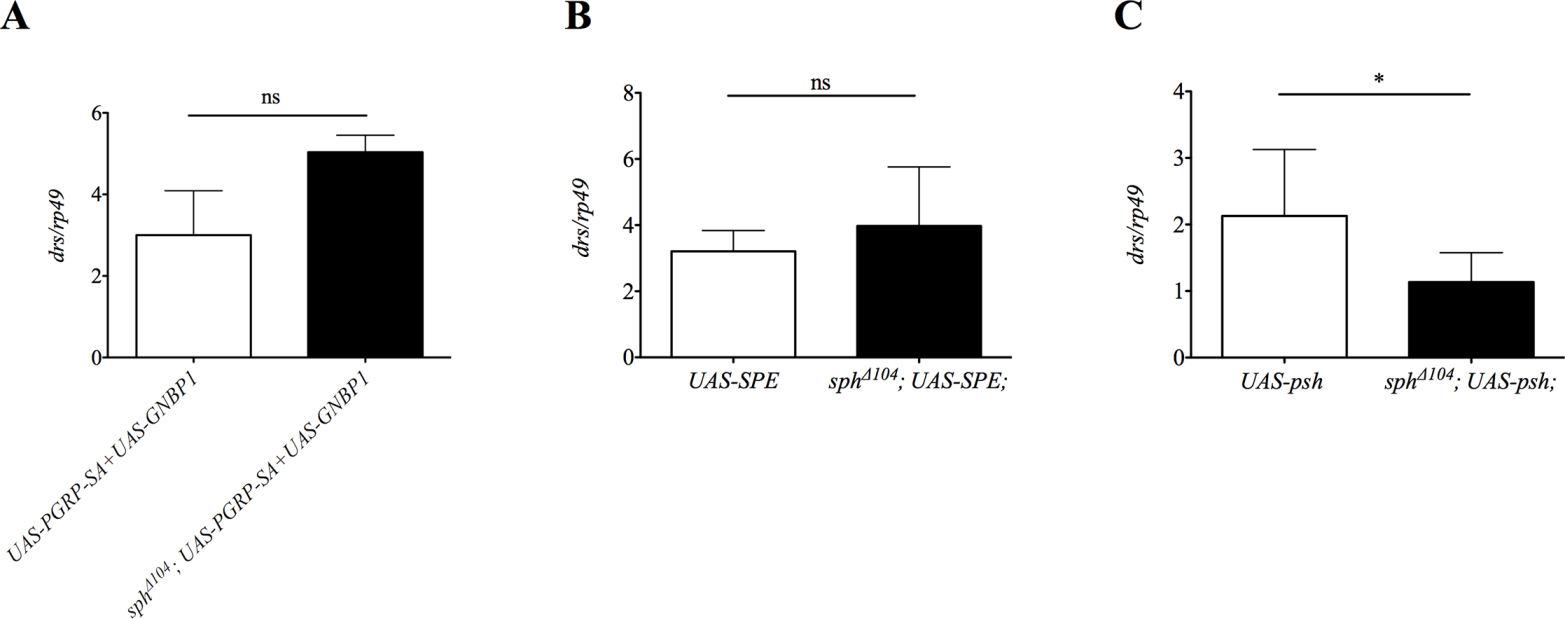
Spheroide is functioning in Psh pathway. *drosomycin* expression normalized to *rp49* after over expression with *yolk>Gal4* (A, B) or with *Act5C>Gal4* (C) (*UAS-psh* vs. *sphe*^***Δ****104*^*; UAS-psh* p<0.0398).

Over expression of serine proteases that lead to proteolytic cleavage of Spz constitutively activates the Toll pathway and induces *drs* expression in the absence of immune challenge. By over expressing the Toll pathway serine proteases in an *sphe* mutant background we assessed the position of Sphe in the cascades. As expected from the phenotype of *sphe* flies, Toll pathway activation after over expression of both PGPR-SA and GNBP1, or of SPE, is not blocked in *sphe* mutant background (Fig 5A and 5B). However, Toll pathway activation after Psh over expression is strongly reduced in *sphe* mutant background (Fig 5C). These observations demonstrate that Sphe is acting downstream of (or at the same level as) Psh in the danger signal cascade.

## Discussion

A previous RNAi screen suggested that Sphe was required for Toll pathway activation [7]. By analyzing the null mutant phenotype of this SPH, we show that Sphe is not required for different kind of infections as previously reported but only after the immune challenge with the pathogenic Gram-positive bacteria *Enterococcus faecalis* and *Staphylococcus aureus*. Furthermore, we demonstrate that Sphe is a component of the danger signal cascade acting downstream (or at the same level) of Psh, and is involved in the sensing of virulence factors (proteases) secreted by these pathogenic bacteria.

The Sphe serine protease homolog has a signal peptide and a trypsin-like protease fold. Within the catalytic triad, the active serine residue is mutated to a glycine residue, blocking the proteolytic activity. Since it has no amidase activity, Sphe cannot directly activate a downstream zymogen.

Recent studies on serine proteases involved in the activation of Toll pathway during embryonic development reported that Gastrulation Defective (GD) forms a complex with Snake (Snk) and Easter (Ea) and that this association is required for the activation of Ea by Snk. When GD is itself activated, its NH_2_-terminal region interacts with sulfated proteins located in the ventral region of the perivitelline membrane. This localization acts to bring Ea and Snk together and promote the ventrally restricted processing of Ea. Surprisingly, this mediation of Snk activity is not dependent on the proteolytic activity of GD but still occurs in GD mutants that lack one of the active catalytic residues [19]. This result establishes that a proteolytically inactive SP can function as a mediator to promote zymogen activation via another SP component of a proteolytic cascade.

Serine protease homologs have been implicated in various physiological processes. In 1991, Hogg et al. reported that a mammalian serine protease homolog, protein Z (PZ), a vitamin K-dependent glycoprotein, binds to thrombin causing its conformational change and its association with phospholipid membrane vesicles. This membrane localization is important during coagulation and clotting as it partitions thrombin to the site of an injury [20]. Later studies of protein crystal structure have demonstrated that PZ functions as a cofactor regulating proteolytic activity of Factor Xa (FXa) on phospholipid vesicles [21]. This is achieved through interaction between the N-terminal domain of PZ and FXa and PZ-dependent Protease Inhibitor (ZPI), which forms a serpin/protease complex with FXa. This interaction is required for the assembly of a protein complex on the phospholipid vesicle surface, which leads to the formation of an effective inhibitory complex containing PZ/FXa/ZPI.

One reported serine protease homolog in *Drosophila*, Masquerade, is necessary during embryonic development to promote and/or stabilize cell-matrix interactions [22]. Homologs of Masquerade in *Tenebrio molitor* and *Holotrichia diomphalia* larvae are required for the proteolytic activation of prophenoloxydase, suggesting a function as a cofactor of the active protease [23]. The *Manduca sexta* serine protease homolog SPH3 is required in the immune response of the moth to infection with Gram-negative bacterium *Photorabdus luminescens*. SPH3 was initially identified as a target for the Repeats-in-toxin (RTX)-metalloprotease, protease A (PrtA), which is secreted by the bacterium. Upon infection, SPH3 is upregulated in both the fat body and hemocytes. RNAi-mediated knockdown of SPH3 increased the susceptibility of moths to infection by *Photorabdus luminescens* [11].

Sphe is involved in the sensing of proteases produced by Gram-positive pathogenic bacteria. We can hypothesize that Sphe is recruited in a complex that mediates the activation of a target SP that could be Persephone on infection with a virulent strain. However, Sphe it is not sensing infection by the entomopathogenic fungus *Beauveria bassiana* nor commercial *Aspergillus oryzea* proteases directly injected in the body cavity even both events induce activation of the Toll pathway through Psh [6] (S1H Fig). Two further SPHs, *sphynx1* and *sphynx2*, were identified in the RNAi screen of Kambris *et al*., together with *sphe*, as putative components of the Toll pathway proteolytic cascade. Since Sphe functions upon infection with specific pathogens, it is likely that Sphynx1 and Sphynx2 might also be involved in the activation of the immune response against other pathogens.

Further investigation is necessary to elucidate the mechanisms by which Sphe is functioning and how it contributes to the sensing of these virulence factors. In addition, characterization of other *Drosophila* SPHs would give insight into their mechanisms of action in other proteolytic cascades.

## Materials and methods

### Fly strains

Stocks were raised on standard cornmeal-yeast agar medium at 25 degrees Celsius, 60% humidity. Flies carrying *UAS-RNAi* transgene against *sphe* (*P{KK112345}VIE-260B*) were obtained from Vienna Drosophila Resource Center (VDRC). Flies carrying Minos transposable element (*Mi{ET1}spheroide*^*MB11555*^) were obtained from Bloomington Stock Center. Flies carrying Minos transposase (*P{hsILMiT}2*.*4*) were obtained from Bloomington Stock Center. Flies that were used as controls in experiments: *w*^*1118*^ (Bloomington Stock Center) or *sphe*^***Δ****11*^are used as wild-type controls, *spz*^*rm7*^ were used as Toll-deficient mutant flies [24], *psh*^*4*^ [25], *grass*^*hrd*^ [6]. Flies with *UAS* constructs used in the study: *UAS-psh* [25], *UAS-SPE* [8], *UAS-grass* [6], *UAS-PGRP-SA*, *UAS-GNBP1* [26]. *UAS-sphe* line was generated in this study: A Myc tag was added at the C-terminal of Spheroide protein using annealed primers IMU938 (5’-GATCCAGGGCGAGCAGAAGCTGATCTCCGAGGAGGACCTGTG-3’) and IMU939 (5’-GATCCACAGGTCCTCCTCGGAGATCAGCTTCTGCTCGCCCTG-3’) cloned in the BamHI site of *CG9675* (*sphe*) cDNA (clone LP05929 from DGRC). The EcoRI-XhoI fragment was inserted in pUAST [27]. Flies carrying different Gal4 drivers (*Act5C* and *Yolk* promoters) were obtained from Bloomington Stock Center. Gal4 driven *RNAi* knockdown was enhanced by incubating three day-old flies for four days at 29°C. *Gal4* driven over expressions were enhanced by incubating three day-old flies for two days at 29°C (epistatic analysis) or four days at 29°C (rescue experiment).

### Microbial strains and infection

For septic injury [28] we used *Micrococcus luteus* (4698), *Enterococcus faecalis* strain OG1RF, protease-deficient strains of *Enterococcus faecalis* (TX5128, TX5243, TX5264) [17], *Staphylococcus aureus* (RN6390), *Enterococcus faecium* DO (TX0016) (obtained from B. Murray). Bacteria were grown in Tryptic Soy Broth (TSB) (*M*. *luteus*) at 30°C or Brain-Heart infusion Broth (BHB) (*E*. *faecalis*, *S*. *aureus* and *E*. *faecium*) at 37°C. Protease-deficient strains TX5128 and TX5243 were cultured with 2mg/ml kanamycin. Bacterial suspensions were prepared from exponential growth phase cultures and diluted to OD600 0.5 in PBS solution for immune challenge except for *M*. *luteus* where a pellet from overnight culture was used.

Natural infection with *Beauveria bassiana* was performed as described [2]. Injection of sublethal doses of commercially available proteases from *Aspergillus oryz*ae (over 500 U/g; P6110; Sigma-Aldrich) was previously described [6].

At least three independent survival experiments were performed. In each experiment and for each genotype, a mix of 20–30 (both males and females) six to eight days-old flies were infected with *E*. *faecalis*, protease-deficient *E*. *faecalis* or *S*. *aureus*, or by natural infection with *B*. *bassiana* at 29°C [27]. The survival data was plotted using GraphPad Prism Software and for statistical analysis we used Log-rank (Mantel-Cox) test.

### Q-RT-PCR analysis

At least three independent experiments were performed in order to analyze transcriptional levels of target genes. In each experiment, the total RNA was extracted from the corresponding genotypes that included a mix of 20–30 (both males and females) six to eight day-old flies using TRI REAGENT® (Molecular Research Center). RNA was extracted from flies 24 hours after (or post-infection, PI) challenged by septic injury with *M*. *luteus*, *E*. *faecalis*, protease-deficient *E*. *faecalis*, *E*. *faecium* and *S*. *aureus*, or 48 hours (or post-infection, PI) after natural infection with *B*. *bassiana* at 29°C, at least three times, independently. Reverse transcription was performed using iScript^TM^ cDNA Synthesis Kit (BIO-RAD). iQ^TM^ SYBR® Green Supermix (BIO-RAD) was used for Quantitative RT-PCR using CFX384™ Real-Time System and CFX Manager 3.0 (BIO-RAD) for data analysis. Student’s t-test was used for statistical analysis using Prism software. *drs*, *sphe* and *rp49* mRNA levels were quantified using these primers: rp49FW (5’-GACGCTTCAAGGGACAGTATCTG-3’), rp49 RV (5’-AAACGCGGTTCTGCATGA-3’), Drom FW (5’-CGTGAGAACCTTTTCCAATATGAT-3’), Drom RV (5-’TCCCAGGACCACGAGCAT-3’, Sph FW (5’-CATTTTGCCGCGTTTGAG-3’), Sph RV (5’-GCATCCGGACTACTATAATCTGAA-3’).

### PCR analysis of deletions

*sphe*^***Δ****11*^ (wild type control), *sphe*^***Δ****49*^ and *sphe*^***Δ****104*^ deletions were generated in this study. Genomic DNA was extracted from single flies using squishing buffer (Tris-HCl pH 8.2 10 mM, EDTA 1 mM, NaCl 25 mM) and proteinase K (200 μg/ml) 30 min at 37 ^o^C and 3 min at 95 ^o^C to inactivate PK. PCR was performed using *Taq* DNA Polymerase (Invitrogen^TM^) with these primers: 682FW (5’-TATGTGGCTGGATGGGGTGAACTT-3’), 4012RV (5’-AATGGGCGGCGGTGACAA-3’) (for *sphe*^***Δ****49*^), 2661RV (5’-TCACGGCCAGGTTGTTGTTCAGAT-3’) (for *sphe*^***Δ****104*^), MinosFW (5’-TCGAATTAATAGTGGTCACTTTTTTT-3’), MinosRV (5’-GTTCGAATTAATAGTGGTTGGGGC-3’), using Tm = 57 ^o^C. PCR with 682FW and 4102RV showed 3331 bp fragment in wild type flies, and a 554 bp fragment in *sphe*^***Δ****49*^ deletion. PCR with 682FW and 2661RV showed 1980 bp fragment in wild type flies and a 725 bp fragment *sphe*^***Δ****104*^ deletion.

## Supporting information

S1 Fig *Spheroide* expression 24 hours PI, normalized to *rp49*. (A). *drosomycin* expression 24 hours PI, normalized to *rp49* after immune challenge with *E*. *faecalis* (*w*^*1118*^ vs. *sphe*^*minos*^ p<0.0458) (B and D) or natural infection with *B*. *bassiana* 48 hours PI (F), or *M*. *luteus* (G) and injection of *A*. *oryzea* proteases (H). Survival rate after immune challenge with *E*. *faecalis* (*w*^*1118*^ vs. *sphe*^*minos*^ p<0.0076) (C) or natural infection with *B*. *bassiana* (E). Schematic representation of deletions obtained by excision of Minos insertion element (I). *w*^*1118*^ wild type flies are used as control (ctrl).
(TIFF)

S2 Fig *drosomycin* expression 24 hours PI. *drosomycin* expression 24 hours PI, normalized to *rp49* after infection with protease-deficient *E*. *faecalis* TX5243 (A) or TX5264 (B). *sphe*^***Δ****11*^ wild type flies are used as control (ctrl).
(TIFF)
