# Supplementary figures and images for: The serine protease homolog spheroide is involved in sensing of pathogenic Gram-positive bacteria
Source: PLoS One. 2017 Dec 6;12(12):e0188339. doi: 10.1371/journal.pone.0188339 (PMC5718610; doi:10.1371/journal.pone.0188339)

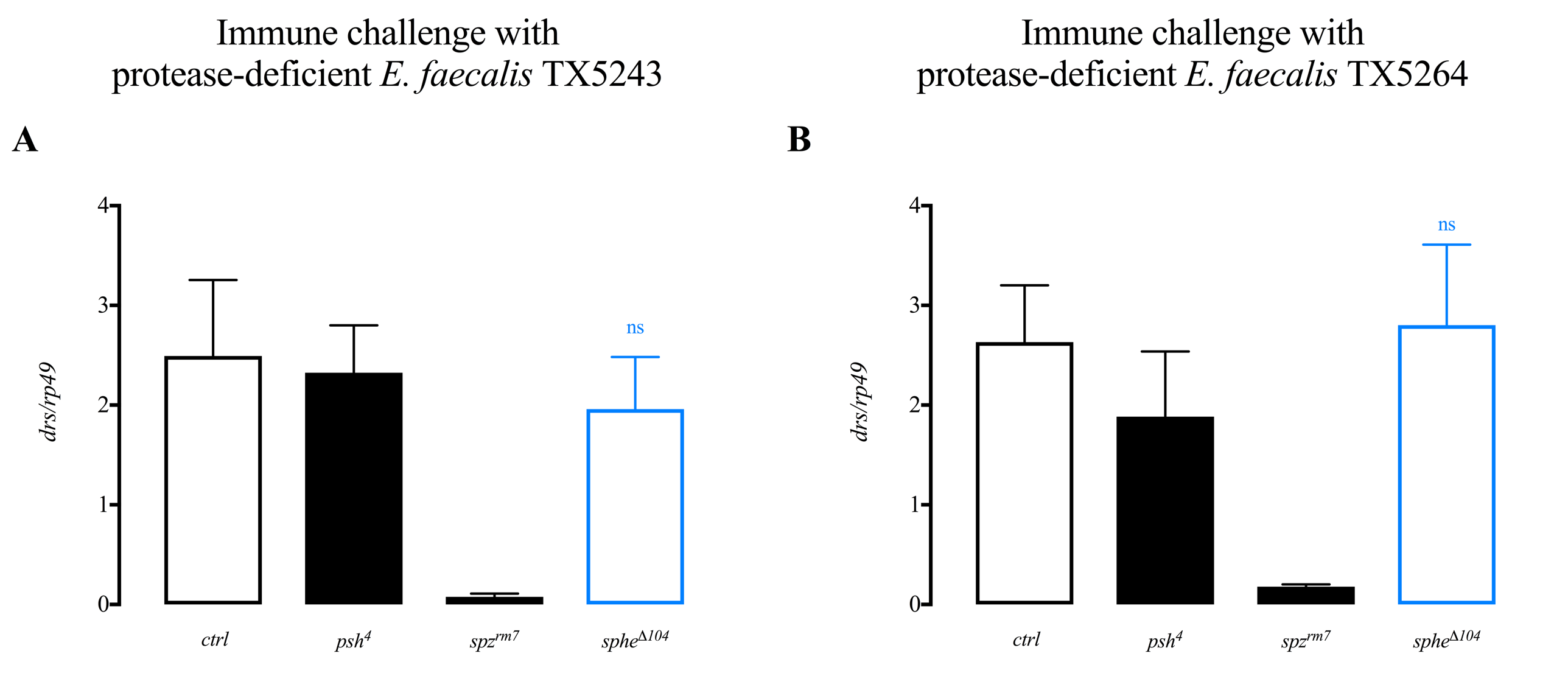

Supplement: S2 Fig — drosomycin expression 24 hours PI, normalized to rp49 after infection with protease-deficient E. faecalis TX5243 (A) or TX5264 (B). spheΔ11 wild type flies are used as control (ctrl). (TIFF) [file pone.0188339.s002.tiff]
